# The antifungal effect of cellobiose lipid on the cells of *Saccharomyces cerevisiae* depends on carbon source

**DOI:** 10.1186/2193-1801-1-18

**Published:** 2012-09-25

**Authors:** Ludmila V Trilisenko, Ekaterina V Kulakovskaya, Tatiana V Kulakovskaya, Alexander Yu Ivanov, Nikita V Penkov, Vladimir M Vagabov, Igor S Kulaev

**Affiliations:** 1 Skryabin Institute of Biochemistry and Physiology of Microorganisms, Russian Academy of Sciences, Pushchino, Moscow Region 142290 Russia; 2 Institute of Cell Biophysics, Russian Academy of Sciences, Pushchino, Moscow Region 142290 Russia

**Keywords:** Cellobiose lipid, Fungicide, *Cryptococcus humicola*, *Saccharomyces cerevisiae*, ATP, Potassium ion, Inorganic polyphosphate, Carbon source

## Abstract

The cellobiose lipid of *Cryptococcus humicola*, 16-(tetra-O-acetyl-β-cellobiosyloxy)-2-hydroxyhexadecanoic acid, is a natural fungicide. Sensitivity of the cells of *Saccharomyces cerevisiae* to the fungicide depends on a carbon source. Cellobiose lipid concentrations inducing the leakage of potassium ions and ATP were similar for the cells grown in the medium with glucose and ethanol. However, the cells grown on glucose and ethanol died at 0.05 mg ml^-1^ and 0.2 mg ml^-1^ cellobiose lipid, respectively. Inorganic polyphosphate (PolyP) synthesis was 65% of the control with 0.05 mg ml^-1^ cellobiose lipid during cultivation on ethanol. PolyP synthesis was not observed during the cultivation on glucose at the same cellobiose lipid concentration. The content of longer-chain polyP was higher during cultivation on ethanol. We speculate the long-chained polyP participate in the viability restoring of ethanol-grown cells after treatment with the cellobiose lipid.

## Background

Some yeasts and mycelial fungi produce glycolipids of various types such as mannosylerythritols, sophorolipids, and cellobiose lipids. They possess multiple biological activities: they act as biosurfactants, facilitate dissolution and consumption of organic hydrophobic compounds, and display fungicidal activity (Kitamoto *et al.*^2002^; Cameotra and Makkar 2004; Golubev 2006; Rodrigues *et al.*^2007^). Cellobiose lipids display antifungal activity against many species of ascomycetous and basidiomycetous yeast and mycelial fungi including know pathogens, *Filobasidiella (Cryptococcus) neoformans* and *Candida albicans* (Puchkov *et al.*^2002^; Kulakovskaya *et al.*^2005^,^2009^; Mimee *et al.*^2005^; Bölker *et al*. 2008; Hammami *et al.*^2010^,^2011^). The broad spectrum of activity, pH and temperature stability allows considering cellobiose lipids as promising compounds for the development of novel fungicides for medical and agricultural applications. So, many studies are now performed in their biochemistry, genetics and possible ecology role (Teichmann *et al.*^2007^*,* Bölker *et al.*^2008^; Hammami *et al.*^2010^,^2011^).

The mechanism of action of cellobiose lipids on yeast cells is based on enhancement of nonspecific permeability of the cytoplasmic membrane, which results in the rapid leakage of ATP and potassium ions from the yeast cells treated with these compounds (Kulakovskaya *et al.*^2005^,^2008^). The glycolipids are surface-active compounds reducing the surface tension of water solutions. Cellobiose lipids of *Cr. humicola* have a high surface activity comparable with that of SDS (Puchkov *et al.*^2002^). The intercalation of glycolipid of *Cr. humicola* into liposomes containing diphytanoylphosphatidylcholine, ergosterol, and phosphatydilserine was demonstrated (Puchkov *et al.*^2002^). These data suggest that the mycocidal effect of cellobiose lipids is associated with its detergent-like properties. Based on these observations and on the electrical measurements on planar phospholipid bilayers, which showed glycolipid-induced membrane permeabilization, it was suggested that the cytoplasmic membrane is the primary target of cellobiose lipid activity (Puchkov *et al.*^2002^).

The fungal species are known to have different sensitivity to cellobiose lipids (Kulakovskaya *et al.*^2005^,^2009^; Mimee *et al.*^2005^). For example, the effective concentrations against basidiomycetes (*Filobasidiella neoformans*) and ascomycetes (*Candida* spp.) are 0.03 mM and 0.1-0.4 mM, respectively (Kulakovskaya *et al.*^2009^). The causes of such difference have not yet been investigated. It is unknown whether cultivation conditions, including those affecting the state of the cytoplasmic membrane, influence the sensitivity of target cells.

The cultivation in ethanol-containing media substantially changes the properties of the cytoplasmic membrane of *Saccharomyces cerevisiae* compared to cultivation in glucose-containing media (Susan *et al.*^1978^; Beaven *et al*. 1982; Mishra and Prasad 1989; Walker-Caprioglio *et al.*^1990^; Herve A *et al.*^1994^; Kubota *et al.*^2004^). During the cultivation on ethanol, the proportion of ergosterol and mono-unsaturated fatty acid residues in cellular phospholipids increases and the fluidity of membrane decreases (Susan *et al.*^1978^; Beaven *et al.*^1982^; Mishra and Prasad 1989; Walker-Caprioglio *et al.*^1990^; Herve A *et al.*^1994^; Kubota *et al.*^2004^). Inorganic polyphosphate (PolyP) is an energy reserve and a stress-protective compound for microbial cells (Kulaev *et al.*^2004^; Rao *et al.*^2009^; Achbergerová and Nahálka 2011). The content and chain length of these bioactive polymers in *Saccharomyces cerevisiae* depend on carbon source (Vagabov *et al.*^2008^). So, cultivation on glucose or ethanol allows obtaining the cells of *S. cerevisiae* which differ in membrane fluidity and PolyP content.

The objective of this work was to compare the sensitivity of *S. cerevisiae* cells grown on glucose and ethanol to the fungicide 16-(tetra-O-acetyl-β-cellobiosyloxy)-2-hydroxyhexadecanoic acid secreted by *Cryptococcus humicola* (Kulakovskaya *et al.*^2009^; Morita *et al.*^2011^). An attempt was made to assess the relationship between PolyP accumulation and sensitivity to cellobiose lipid.

## Results and discussion

**Figure 1:**
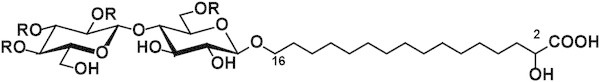
**The structure of cellobiose lipid secreted by*Cryptococcus humicola*strain 9-6. R - acetate.**

The cellobiose lipid preparation used in the work was obtained from the culture liquid of *Cr. humicola* strain 9-6 (All-Russian Collection of Microorganisms). Mass spectrometry shows that the major component of the preparation has a molecular mass 781 kDa. This compound is a 16-(tetra-O-acetyl-β-cellobiosyloxy)-2-hydroxyhexadecanoic acid (cellobiose lipid) according to earlier data (Puchkov *et al.*^2002^; Kulakovskaya *et al.*^2009^) (Figure 1).

The cells of *S. cerevisiae* proved to have different survival capacities at the same cellobiose lipid concentrations depending on the carbon source used. The cells grown on glucose died at a concentration of 0.05 mg ml^-1^, while the cells grown on ethanol died at 0.2 mg ^-1^ (Table 1).

**Table 1:** **The viability of the cells of*Saccharomyces cerevisiae*grown in the media with glucose and ethanol treated with cellobiose lipid**

Cellobiose lipid, mg ml^-1^	Cell viability, % of control	
	Glucose grown cells	Ethanol grown cells
0	100	100
0.025	60 ± 12	-
0.034	11 ± 1.1	-
0.050	4 ± 1.7	100
0.100	0.2	16 ± 5.1
0.200	0.1	2 ± 0.5
0.400	0	1.5
0.800	0	0

- not assayed.

Cellobiose lipid shows fungicidal activity in acidic medium, where it is a weak acid due to dissociation of the carboxyl group (Puchkov *et al.*^2002^; Kulakovskaya *et al.*^2009^). The average values of electrokinetic potential (EKP) were calculated to be 18.8±1.2 and 23.6±3.0 mV for the cells of *S. cerevisiae* grown on glucose and ethanol, respectively. It is probable that the high negative surface charge decreases the binding of negatively charged molecules of the fungicide.

**Figure 2:**
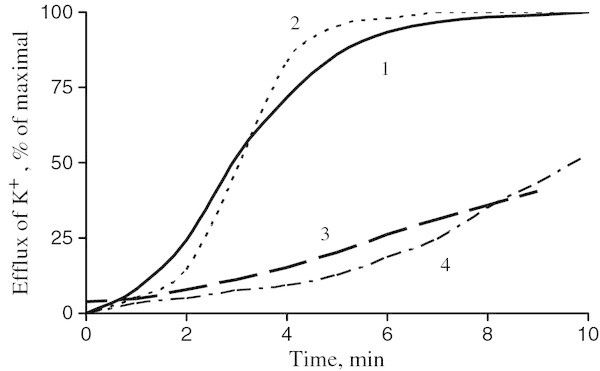
**Potassium leakage from the cells of*Saccharomyces cerevisiae*treated with cellobiose lipid of*Cr. humicola*: 1 and 3 - the cells were grown in the medium with glucose, 2 and 4 - the cells were grown in the medium with ethanol.** The concentration of cellobiose lipid was 0.24 mg ml^-1^ (1 and 2) and 0.12 mg ml^-1^ (3 and 4).

One of the known criteria of yeast cytoplasmic membrane integrity damage is the leakage of potassium ions into the medium (Kulakovskaya *et al.*^2008^; Shirai *et al.*^2009^). The effective cellobiose lipid concentrations inducing K^+^ leakage were not different for the cells of *S. cerevisiae* grown on both carbon sources (Figure 2). Consequently, the high stability of *S. cerevisiae* cells grown on ethanol cannot be explained solely by intensification of the barrier functions of the membrane.

**Figure 3:**
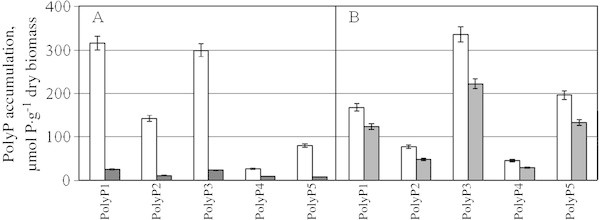
**The accumulation of different fraction of inorganic polyphosphate (PolyP) in the cells of*Saccharomyces cerevisiae*for 30 min of cultivation in phosphate-rich medium with glucose (A) and ethanol (B).** (□) - control, (■) - in the presence of cellobiose lipid, 0.05 mg ml^-1^.0.

The energy of the phosphoester bond in PolyP is similar to that of ATP. PolyP is known to be a factor of microbial cell resistance to stress conditions (Kulaev *et al.*^2004^; Rao *et al.*^2009^; Achbergerová and Nahálka 2011). Hence, the effect of cellobiose lipid on the PolyP, P_i_ and ATP content in the cells has been studied. The experiments were performed under the conditions of PolyP synthesis. The cells with the PolyP content of 50–65 µmole P/g dry biomass (not shown) were cultivated in the complete medium for 30 min. Then PolyP fractions with different chain lengths were extracted. PolyP accumulation was observed in both media: with glucose and with ethanol (Table 2, Figure 3). PolyP synthesis was almost completely suppressed by cellobiose lipid in the medium with glucose (Figure 3, Table 2) but only by 35% lower in the medium with ethanol.

**Table 2:** **The effect of cellobiose lipid on the content of P**_**i**_**, PolyP and ATP (µmol g**^**-1**^**dry biomass) in the cells of*S. cerevisiae*under PolyP synthesis: the cells after P**_**i**_**starvation were cultivated in the complete medium with 10 mM P**_**i**_**for 30 min**

Culture condition	Glucose ethanol		Glucose ethanol		Glucose ethanol	
	P_i_		PolyP		ATP	
Control	45	41	870	825	5.1	13
Cellobiose lipid, 0.05 mg ml^-1^	3.4	20	80	560	1.2	6.0

The chain length of PolyP of different fractions determined by electrophoresis in PAAG did not depend on the carbon source in the presence and absence of cellobiose lipid (Table 3). The content of longer-chain fractions was higher during cultivation on ethanol (Figure 3 and Table 3). During the cultivating on glucose, the P_i_ and ATP content decreased in the presence of cellobiose lipid much more than during the cultivation on ethanol (Table 2).

**Table 3:** **Polyphosphates in*Saccharomyces cerevisiae*after 30 min cultivation in the media with glucose or ethanol: the average chain length and proportion of PolyP of different fractions**

Fraction	Average chain length (n)	% of total PolyP content	
		Glucose	Ethanol
PolyP1	15	37	20
PolyP2	25	16	9
PolyP3	65	35	41
PolyP4	75	3	6
PolyP5	>200	9	24

The cells after P_i_ starvation were cultivated in the complete medium with 10 mM P_i_.

We have also determined the effect of cellobiose lipid on ATP leakage from cells in phosphate-citrate buffer (pH 4.0). The effective glycolipid concentrations were different for the cells grown on glucose and ethanol (Table 4). The addition of glucose decreased the effective concentration of the fungicide only for glucose-grown cells. This effect was not observed in the cells grown on ethanol.

**Table 4:** **The concentrations of cellobiose lipid (mg ml**^**-1**^**) causes the maximal leakage of ATP in phosphate-citrate, pH 4.0 from*S. cerevisiae*cells**

Incubation medium	Cells grown in medium with glucose	Cells grown in medium with ethanol
	The concentration of cellobiose lipid	
0.04 M citrate-phosphate, pH 4.0	0.6	0.6
0.04 M citrate-phosphate, pH 4.0, 2% glucose	0.15	0.6

The sensitivity of the cells of *S. cerevisiae* to 16-(tetra-O-acetyl-β-cellobiosyloxy)-2-hydroxyhexadecanoic acid depends on the carbon source used for cell cultivation. The cells grown on ethanol are more resistant to this membrane damaging fungicide. It is probably due to the increase of the negative charge of cell surface (EKP) during cultivation on ethanol and to the change in membrane lipid composition. However, the effects of cellobiose lipid on potassium leakage were similar for the cells grown in the media with glucose or ethanol. It suggests the existence of additional factors increasing the resistance of yeast grown on ethanol to cellobiose lipid. We speculate the long-chained polyP participate in the restoring of viability of ethanol-grown cells after treatment with the cellobiose lipid.

## Conclusion

The sensitivity of yeast cells to antifungal cellobiose lipids depends on culture conditions especially on carbon source. The peculiarities of growth conditions of target microorganisms should be taken into account when assessing effective concentrations of these new fungicidal compounds.

## Materials and methods

### Strains and growth conditions

The yeast *Saccharomyces cerevisiae* strain VKM Y-1173 was grown in a shaker in the Reader medium with 0.2% yeast extract, 2% glucose (120 r.p.m.), or 1% ethanol (200 r.p.m.). The medium contained (g l^-1^): (NH_4_)_2_SO_4_, 3; MgSO_4_, 0.7; Ca(NO_3_)_2,_ 0.4; NaCl, 0.5; KH_2_PO_4_, 1; K_2_HPO_4_, 0.1; (NH_4_)_2_SO_4_^.^ FeSO_4_^.^ 6H_2_O, 0.00025; and trace elements (Vagabov *et al.*^2000^).

Polyphosphate (PolyP) biosynthesis was studied using the cells with low PolyP levels grown in a phosphate-free medium as described in (Vagabov *et al.*^2000^). Then the cells were cultivated in complete medium for 0.5 h. Biomass samples were harvested at 3000 g for 10 min, washed twice with distilled water at 4°C, and used for PolyP extraction. Dry cell mass was determined after drying cell aliquots at 85°C under vacuum.

### Purification of cellobiose lipids

The cellobiose lipid of the yeast *Cryptococcus humicola* 9-6 (All-Russian Collection of Microorganisms, VKM) was obtained as described (Kulakovskaya *et al.*^2009^). After the cultivation, the culture supernatant was separated by centrifugation at 5000 g for 40 min, filtered through a Whatman glass fiber filter GF/A from Sigma-Aldrich Rus (Moscow, Russia), and lyophilized. The residue was extracted with methanol for four to five days at 5°C and filtered. The filtrate was evaporated at 50°C, and the resulting product was suspended in deionized water. The suspension was kept for 24 h at 5°C, and the resulting precipitate was separated by filtration through a glass filter, washed twice with cooled deionized water, and dissolved in methanol. The concentration of glycolipids was determined by weighing after methanol evaporation. In the course of purification, the antifungal activity was assayed by placing the aliquots of preparations on glucose-peptone agar (GPA) containing 0.5% glucose, 0.2% yeast extract, 0.25% peptone, 2% agar, 0.04 M citrate-phosphate buffer, pH 4.0 and inoculated with *S. serevisiae*.

### ESI-MS analysis

The ESI-MS spectra were recorded with a Finnigan MAT LCQ (San Jose, CA, USA) mass spectrometer as described earlier for positive ions (Kulakovskaya *et al.*^2005^,^2009^. For direct (syringe) inlet, the methanol solution of a sample was injected at 10 µL/min. MS spectra were measured in positive mode.

### Inorganic polyphosphate (PolyP) assay

Five separate polyP fractions differ in the chain length were obtained from *Saccharomyces cerevisiae* cells as described in (Vagabov *et al.*^2000^). Acid-soluble polyphosphates (PolyP1) were extracted with 0.5N HClO_4_. Salt-soluble polyphosphates (PolyP2) were extracted with saturated NaClO_4_ solution. Two fractions of alkali-soluble polyphosphates (PolyP3 and PolyP4) were extracted with the weak NaOH solution (pH was adjusted to 9–10) and 0.05 M NaOH (pH 12), respectively. All extractions were performed twice at 0°C under stirring for 10 min. The PolyP contents in the fractions PolyP1, PolyP2, PolyP3 and PolyP4 were quantified as a difference in P_i_ amounts before and after hydrolysis of the samples in 1N HCl for 10 min at 100°C. The level of PolyP5 fraction was determined by treating residual material with 0.5N HClO_4_ at 90°C twice for 20 min and assaying the released P_i_. P_i_ was determined according to (Vagabov *et al.*^2000^). The data in the tables and figures are the average values of three experiments.

The chain length of PolyP from different fractions was determined by electrophoresis in polyacrylamide gel according to (Kumble and Kornberg 1995). For PolyP5 electrophoresis, residual biomass was extracted with distilled water for 12 h (Vagabov *et al.*^2008^). PolyP standards with the average chain lengths of 15, 25, 45, 75 phosphate residues were from Sigma (St Louis, USA), and with the average chain lengths of 208 phosphate residues were from Monsanto (St Louis, USA).

### ATP assay

The ATP content in the cells was assayed after treating biomass samples with dimethylsulfoxide (0.2 ml / 25–50 mg of wet biomass). The effects of cellobiose lipids on ATP leakage from the cells was assayed as described (Kulakovskaya *et al.*^2003^). ATP was assayed by the luciferin-luciferase method using a Sigma assay kit and a LKB 1250 Luminometer (Sweden).

### Potassium ion leakage

The leakage of K^+^ from the yeast cells was registered with a K^+^-selective electrode (Orion, USA). The measurements were made in a thermostatically controlled 2.5 ml cell at 25°C under stirring. The measuring medium containing 0.01 M citrate-phosphate buffer, pH 4.0, was injected with 50 µl of cell suspension to a final cell concentration of 6–6.5 · 10^8^ ml^-1^. The maximum quantity of K^+^ found in the medium was taken as 100%.

### Measurement of EKP (electrokinetic potential)

The cells were suspended in 0.01 M citrate buffer, pH 4.0, to a concentration of 10^7^ – 5 × 10^7^ cells ml^-1^. The EKP of yeast cells was measured with a Zetasizez nano ZS (Malvern, Great Britain) by the method of laser Doppler spectroscopy at 25°C. The average EKP value was calculated from three repeated measurements in each population of yeast cells.

### The assay of cell viability

Yeast cell viability assay was performed as follows. The starting cell suspension was diluted in distilled water (1:100). Then the cells were treated with different cellobiose lipid concentrations (0.025 to 0.8 mg ml^-1^). The incubation mixture contained 0.5 ml of 0.04 M citrate-phosphate buffer, pH 4.0, and 0.1 ml of cell suspension. The mixture without cellobiose lipid was used as a control (100% viability). After the treatment, the cells were incubated at 30°C for 1 h. Then the cell suspensions were diluted in the citrate-phosphate buffer to different ratios and deposited on Petri dishes. The Petri dishes were incubated at 28°C for 3 days and the number of colonies was calculated.

All experiments were performed in triplicate. The biochemicals except those which are listed separately were obtained from Sigma-Aldrich-Rus (Moscow, Russia).

## Abbreviations

PolyP: Inorganic polyphosphate
16-(tetra-O-acetyl-β-cellobiosyloxy)-2-hydroxyhexadecanoic acid: Cellobiose lipid.
